# Enhanced climate instability in the North Atlantic and southern Europe during the Last Interglacial

**DOI:** 10.1038/s41467-018-06683-3

**Published:** 2018-10-12

**Authors:** P. C. Tzedakis, R. N. Drysdale, V. Margari, L. C. Skinner, L. Menviel, R. H. Rhodes, A. S. Taschetto, D. A. Hodell, S. J. Crowhurst, J. C. Hellstrom, A. E. Fallick, J. O. Grimalt, J. F. McManus, B. Martrat, Z. Mokeddem, F. Parrenin, E. Regattieri, K. Roe, G. Zanchetta

**Affiliations:** 1 Environmental Change Research Centre, Department of Geography, University College London, London WC1E 6BT, UK; 2 School of Geography, The University of Melbourne, Melbourne, VIC 3053 Australia; 3 Laboratoire EDYTEM UMR CNRS 5204, Université Savoie Mont Blanc, 73376 Le Bourget du Lac, France; 4 Department of Earth Sciences, University of Cambridge, Cambridge CB2 3EQ, UK; 5 Climate Change Research Centre and ARC Centre of Excellence for Climate System Science, University of New South Wales, Sydney, NSW 2052 Australia; 6 Department of Earth and Planetary Sciences, Macquarie University, Sydney, NSW 2109 Australia; 7 School of Earth Sciences, The University of Melbourne, Melbourne, VIC 3010 Australia; 8 Scottish Universities Environmental Research Centre, East Kilbride G75 0QF, UK; 9 Department of Environmental Chemistry, Spanish Council for Scientific Research (CSIC), Institute of Environmental Assessment and Water Research (IDAEA), Barcelona 08034, Spain; 10 Lamont-Doherty Earth Observatory, Columbia University, Palisades, NY 10964 USA; 11 CNRS, IRD, IGE, Université Grenoble Alpes, 38000 Grenoble, France; 12 Dipartimento di Scienze della Terra, University of Pisa, Pisa 56126, Italy

## Abstract

Considerable ambiguity remains over the extent and nature of millennial/centennial-scale climate instability during the Last Interglacial (LIG). Here we analyse marine and terrestrial proxies from a deep-sea sediment sequence on the Portuguese Margin and combine results with an intensively dated Italian speleothem record and climate-model experiments. The strongest expression of climate variability occurred during the transitions into and out of the LIG. Our records also document a series of multi-centennial intra-interglacial arid events in southern Europe, coherent with cold water-mass expansions in the North Atlantic. The spatial and temporal fingerprints of these changes indicate a reorganization of ocean surface circulation, consistent with low-intensity disruptions of the Atlantic meridional overturning circulation (AMOC). The amplitude of this LIG variability is greater than that observed in Holocene records. Episodic Greenland ice melt and runoff as a result of excess warmth may have contributed to AMOC weakening and increased climate instability throughout the LIG.

## Abstract (web summary)

It is important to establish a baseline for natural climate variability under relatively warm conditions. Here we show that the Last Interglacial in the North Atlantic and southern Europe was characterized by enhanced climate instability relative to the pre-industrial Holocene.

## Introduction

The Last Interglacial (LIG; 129–116 thousand years ago (ka)) was characterized in its earlier part by strong positive summer insolation and temperature anomalies at high northern latitudes, amplified by ocean, sea ice and land ice, and vegetation feedbacks^1,2^. Attendant sea-level rise is estimated to have been ~6–9 m above present, with 0.6–3.5 m derived from Greenland ice-sheet (GrIS) melting^3^. Although several North Atlantic and European records indicate the presence of LIG climate instability, the number, timing, and geographic extent of millennial/centennial-scale climate oscillations remain unclear. Sea-surface temperature (SST) fluctuations have been detected in the Nordic Seas^4^, and lithological variations in subpolar North Atlantic cores have indicated multiple incursions of drift ice^5^. Isotopic, faunal and ice-rafted detritus (IRD) analyses have revealed the presence of a series of moderate surface-cooling events and decreases in benthic foraminiferal δ^13^C (refs. ^6–11^). However, the relationship between ocean surface variability and changes in deep-water circulation remains ambiguous. LIG climate instability has been detected in European pollen and speleothem records^12–18^, but correlation uncertainties complicate an assessment of how terrestrial events are related to ocean changes. Compounding the ambiguity, the Greenland NEEM LIG record, reconstructed from a folded ice core, shows a relatively smooth surface temperature profile with gradual cooling following early peak conditions, but also indicates multiple episodes of extensive ice surface melting^19^.

Here we bypass correlation uncertainties by linking terrestrial and marine records through joint pollen and ocean proxy analyses in a deep-sea core on the Portuguese Margin. A key to this is the geographical setting of the area where the combined effects of the Tagus River and a narrow continental shelf lead to the rapid delivery of terrestrial material, including pollen, to the deep-sea environment. Previous joint marine–terrestrial analyses on the Portuguese Margin^20^ led to a re-evaluation of the LIG duration in southern Europe, but the sampling resolution (~500 years) was not sufficient to establish the presence of abrupt events. We return to the same area and generate palaeoceanographic proxies and pollen records from the same sample depths at ~100-year resolution in core MD01-2444 (see Methods). To produce an independent timescale of interglacial changes, we use a new, stacked δ^18^O_speleothem_ record from Antro del Corchia, a large cave system in the Alpi Apuane karst, Italy, comprising four individual stalagmites from a single chamber, and anchored in time by 87 uranium–thorium (U–Th) ages (see Methods). The cave receives most of its recharge rainfall via westerly air masses crossing the North Atlantic, and previous work has shown a close connection between changes in surface-ocean and atmospheric conditions on the Iberian Margin and the amount of rainfall reaching the site^21^. Combining cave and ocean data in this way provides a stratigraphic lattice, linking changes in North Atlantic and Southern European conditions and placing them on a detailed chronological framework. We then compare our records with two of the most detailed marine sequences in the northern North Atlantic, containing evidence for changes in ocean circulation during the LIG. Finally, to explore the mechanisms and spatial distribution of climate variability, we perform a suite of experiments under LIG boundary conditions with the LOVECLIM Earth system model and with the atmospheric component of the NCAR Community Earth System Model (CESM, see Methods). We found that the LIG was punctuated by a series of multi-centennial arid events in southern Europe and cold water-mass expansions in the North Atlantic, consistent with low-intensity disruptions of the overturning circulation. Our results suggest that the LIG was characterized by enhanced climate instability relative to the pre-industrial Holocene.

## Results and discussion

### Linking terrestrial and marine records

**Fig. 1:**
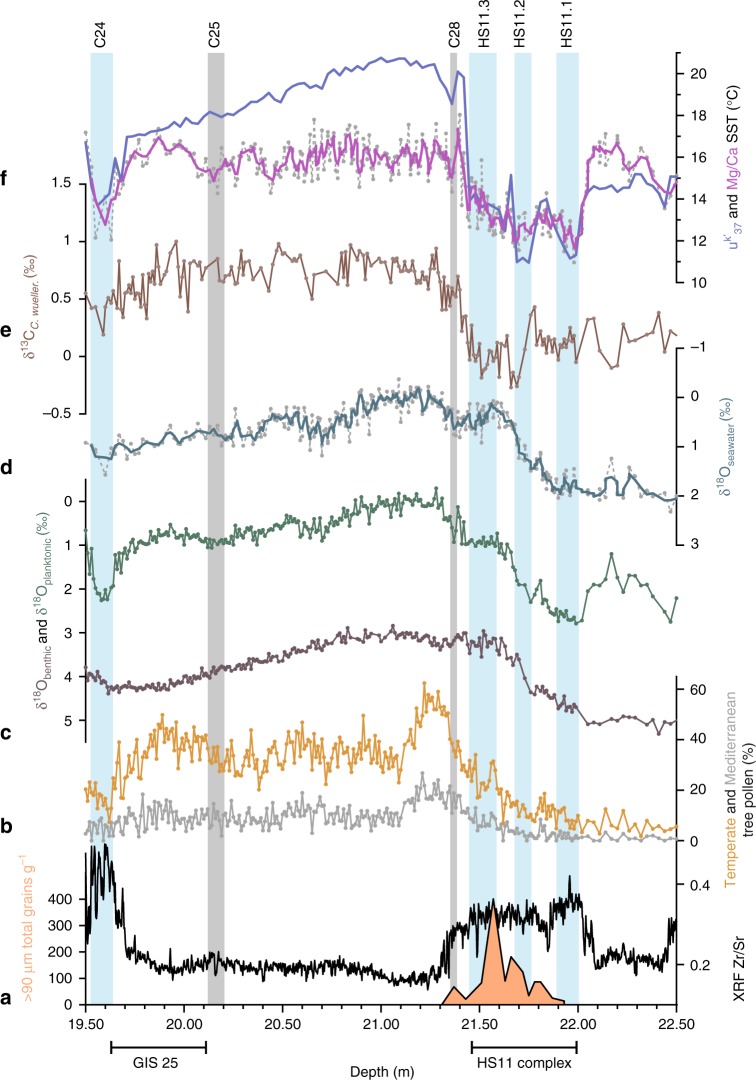
MD01-2444 LIG records plotted against depth. **a** XRF Zr/Sr record; ice-rafted detritus (IRD) counts for the interval 21.3–21.95 m only^25^. **b** Mediterranean and temperate (Mediterranean + Eurosiberian) tree pollen percentages (see Methods). **c** Planktonic (*G. bulloides*) and benthic foraminiferal δ^18^O. **d** Seawater δ^18^O, derived from *G. bulloides* δ^18^O and Mg/Ca SST. **e** δ^13^C of epibenthic species *C. wuellerstorfi*. **f** Alkenone-based u^k′^_37_ (ref. ^24^) and *G. bulloides* Mg/Ca SST. Vertical bars indicate events HS11.1, HS11.2, HS11.3, C28, C25 and C24

Figure 1 shows that high values in the X-ray fluorescence (XRF) Zr/Sr ratio (reflecting the relative proportion of detrital and biogenic sediment supply) at 22–21.45 and 19.63–19.52 m demarcate Heinrich Stadial 11 (HS11)^22^ and cold water event 24 (C24), respectively^23^. Closer inspection suggests that HS11 contains three cold events (HS11.1, HS11.2, HS11.3) as indicated by declines in alkenone^24^ and *Globigerina bulloides* Mg/Ca SST, and δ^13^C of epibenthic species *Cibicidoides wuellerstorfi* (δ^13^C_*C. wueller*._). HS11.2 and HS11.3 are characterized by high IRD values, δ^13^C_*C. wueller*._ minima, and low δ^18^O_planktonic_ and seawater δ^18^O (δ^18^O_sw_) values, pointing to large meltwater pulses and Atlantic meridional overturning circulation (AMOC) weakening, in agreement with previous results^25^. The onset of the interglacial at the end of HS11.3 is marked by 5–6 °C rise in SST and increases in δ^13^C_*C. wueller*._ and temperate tree pollen values. A brief climatic reversal at ~21.35 m, indicated by a small IRD peak and decreases in SST, δ^13^C_*C. wueller*._ and temperate tree pollen, is correlative with event C28 in North Atlantic records^7–9^. This is followed by peak interglacial values in temperate tree pollen, including a peak in Mediterranean taxa, indicating increased summer temperature and evaporation and enhanced winter precipitation, in line with evidence from southern Europe^26^. The section 21.10–20.15 m contains reduced variability, but is punctuated by episodic decreases in temperate tree pollen values and small increases in δ^18^O_planktonic_, which are not always observed in our SST records. This could arise from habitat biases that limit the proxy-carrier’s ability to record moderate temperature changes^27^, as well as the effects of upwelling from May/June to September/October along the Portuguese Margin^28^. A small increase in XRF Zr/Sr values coupled with lower Mg/Ca SST and temperate tree pollen values and higher δ^18^O_planktonic_ between 20.20 and 20.12 m is correlative with event C25 in North Atlantic records^7–9^. The section 20.12–19.70 m, characterized by increased Mg/Ca SST and temperate tree pollen values and lower δ^18^O_planktonic_, corresponds to Greenland InterStadial GIS 25c (GIS nomenclature follows ref. ^29^); the second part of this interval has higher Mediterranean tree pollen values, coeval with higher CH_4_ values within GIS 25c (ref. ^30^). At 19.70 m, Mg/Ca SST, δ^18^O_planktonic_, temperate tree pollen and XRF Zr/Sr values begin to shift to stadial conditions interrupted by a brief warm reversal, similar to the sequence of events GIS 25b and GIS 25a observed in Greenland ice-core records^29,30^.

**Fig. 2:**
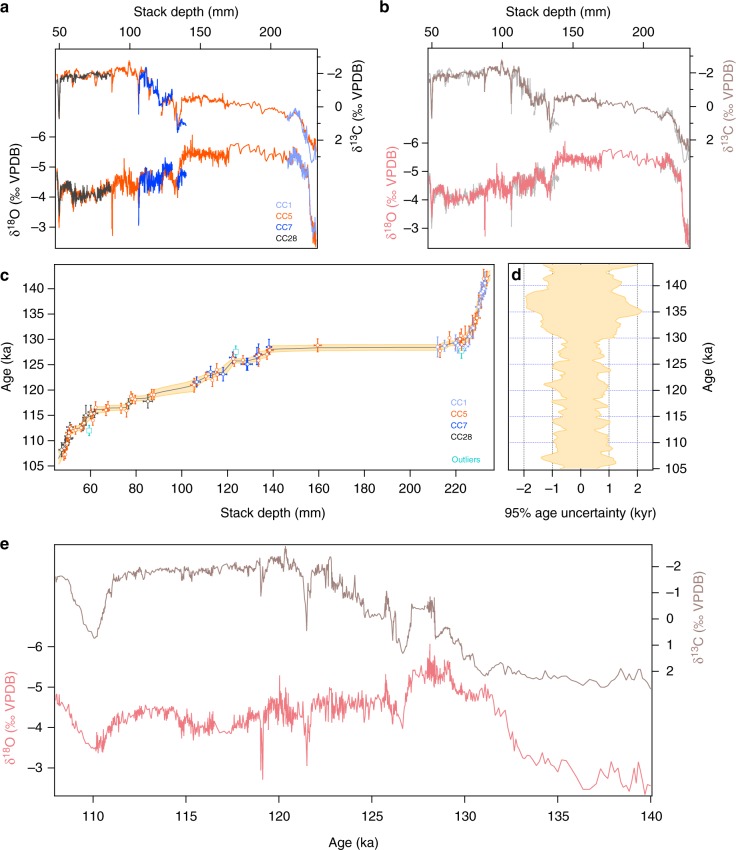
Antro del Corchia speleothem stack and age model. **a** Individual Corchia speleothem δ^13^C and δ^18^O series tuned to a common (stack) depth scale. **b** The composite Corchia speleothem δ^13^C (brown) and δ^18^O (pink) series plotted on the stack depth scale. The grey background curves are the individual series from **a**. **c** The Corchia stack age model based on 90 U–Th ages (colour coded by speleothem). The cyan symbols are three outlying ages rejected from the modelling (see Methods). The age model is based on the remaining 87 ages. The pale yellow envelope represents the 95% model-age uncertainties. Horizontal bars represent 100% sampling errors, while the vertical errors bars are the 95% uncertainties on the individual U–Th age determinations. **d** The 95% age-uncertainty envelope vs. age derived from the age model shown in **c**. **e** The final composite Corchia δ^18^O and d δ^13^C time series based on the age model shown in **c**

Compared to previous work on the LIG in Corchia Cave^21^, the new speleothem stack provides a continuous, higher-resolution and intensively dated record of the interval 140–108 ka (Fig. 2). The stack was produced by synchronizing previously published individual speleothem stable isotope records^21,31–33^ to a common depth scale (Fig. 2a), assigning the U–Th ages from individual speleothems to their corresponding position on this same depth scale, then producing a single depth-age model (Fig. 2c; see Methods and Supplementary Figs. 1, 2 for further details). In addition to providing a robust test of replication amongst coeval samples, the advantage of stacking is that it uses all speleothem ages and their ±2*σ* errors, which significantly increases the age density and therefore yields a depth-age model with markedly reduced age uncertainties (Fig. 2d) compared to the age model of any single speleothem (see Supplementary Fig. 2). The final stacked isotope time series (Fig. 2e) utilizes the highest resolution data from the individual speleothems (Fig. 2b). This yields a continental isotope time series for the LIG of compelling detail, displaying a series of millennial-to-centennial-scale isotopic excursions that are evident in both isotope ratios.

**Fig. 3:**
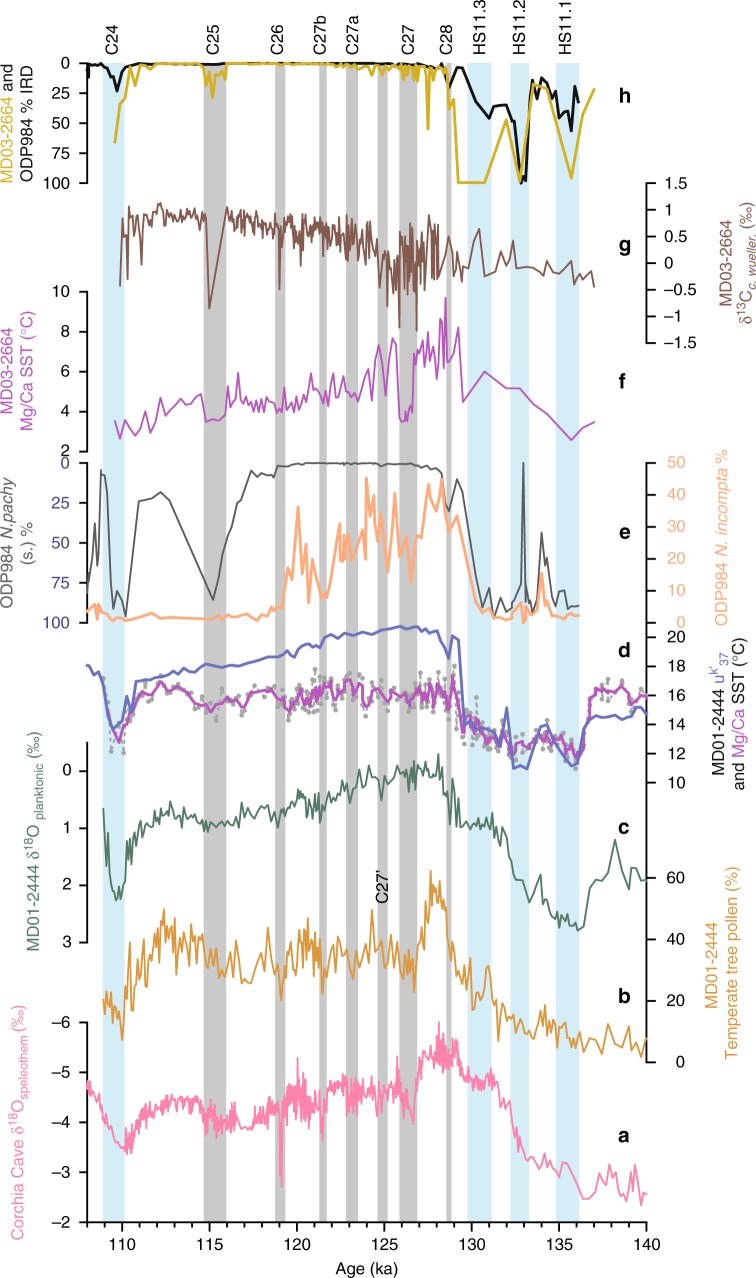
Comparison of LIG paleoclimate records. **a** Corchia Cave stacked δ^18^O_speleothem_ series. **b–d** MD01-2444 temperate tree pollen, δ^18^O_planktonic_, alkenone ^uk'^_37_ and Mg/Ca SST. **e** ODP984 *Neogloboquadrina pachyderma* (s) (inverse scale), *N. incompta* percentages^9^. **f**, **g** MD03-2664 Mg/Ca SST and δ^13^C_*C. wueller.*_ (refs.^10, 39^). **h** ODP984 (ref. ^9^) and MD03-2663 (ref. ^39^) IRD percentages. All records plotted on the Corchia Cave timescale (see Methods). Light blue vertical bars indicate events HS11.1, HS11.2, HS11.3 and C24; grey bars denote cold events C28, C27, C27a, C27b, C26 and C25 (refs. ^6–9^), and one identified on the Portuguese Margin, labelled C27′

Examination of the MD01-2444 and Corchia archives (Fig. 3a, b) reveals a striking similarity between the temperate tree pollen and δ^18^O_speleothem_ records and this forms the basis for transferring the Corchia U–Th timescale to the MD01-2444 sequence (see Methods and Supplementary Fig. 3), on the premise that rainfall amount in southern Europe exerts a dominant control over both the composition of vegetation and the isotopic signature of Corchia speleothems^21,31,34^. Lower δ^18^O_sw_ values (Fig. 1d) associated with the HS11.2 and HS11.3 meltwater pulses suggest that at least part of the more negative δ^18^O_speleothem_ signal at that time (Fig. 3a) is related to changes in surface hydrography and their influence on the isotopic signature of moisture advected across the Mediterranean^35^. Further shifts in δ^18^O_speleothem_ and also temperate tree pollen and SST (Fig. 3a, b, d) mark the onset of the LIG at ~129.3 ka. After the C28 reversal, the speleothem and pollen records show a climatic optimum at ~128 ka characterized by increased (winter) precipitation. After that, δ^18^O_speleothem_ and temperate tree pollen records indicate a series of low-amplitude decreases in rainfall amount in Iberia and Italy, centred at ~126.4, 124.9, 123.1, 121.4, 119.1, 117.2 and 115.3 ka. The replication of these events as increases in δ^13^C_speleothem_ (Fig. 2e) suggests that these rainfall reductions were probably associated with regional cooling, which would decrease the contribution of isotopically depleted, soil-derived biogenic CO_2_ reaching the cave chamber^32,33,36^. The duration of these events is 0.6–0.8 kyr, apart from the one centred at 115.3 ka, which lasts 1.5 kyr and contains internal variability. Intra-interglacial oscillations are also observed in speleothem, pollen and lithological records in SW France, Italy, Greece and eastern Turkey^13–15,17,37^, pointing to a pervasive variability in the hydrological cycle across southern Europe and the Near East.

**Fig. 4:**
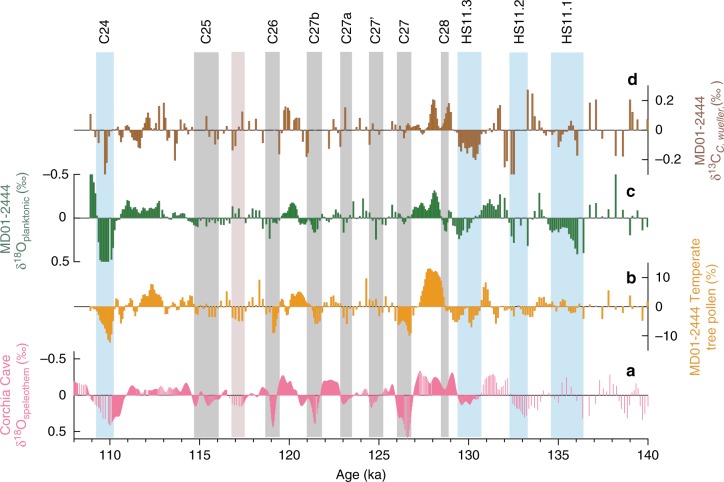
Comparison of intra-interglacial aridity events and ocean changes. **a** Detrended and smoothed Corchia Cave stacked δ^18^O_speleothem_ series. **b** Detrended and smoothed MD01-2444 temperate tree pollen. **c** Detrended and smoothed MD01-2444 δ^18^O_planktonic_. **d** Detrended and smoothed MD01-2444 δ^13^C_*C. wueller*._ (see Methods). Records plotted on the Corchia Cave timescale. Light blue vertical bars indicate events HS11.1, HS11.2, HS11.3 and C24; grey bars denote interglacial arid events (defined in Corchia Cave δ^18^O_speleothem_ and MD01-2444 temperate tree pollen records) that show coupled pollen and δ^18^O_planktonic_ changes and light pink bar denotes an aridity event associated with negative rather than positive δ^18^O_planktonic_ values (see Methods)

We then explore whether the aridity events defined in the Corchia Cave δ^18^O_speleothem_ and MD01-2444 pollen records are associated with ocean changes on the Portuguese Margin. We detrend and smooth our records to examine whether decreases in temperate tree pollen values during these events are accompanied by increases in δ^18^O_planktonic_ values in two or more stratigraphically successive samples (see Methods and Fig. 4). As the analyses were undertaken on the same samples in MD01-2444, the comparison is independent of chronological uncertainties. We also inspect the δ^13^C_*C. wueller*._ record throughout the entire 108–140 ka interval, but note that *C. wuellerstorfi* is not continuously present within the interglacial. HS11.1–11.3 and C24 are clearly indicated by changes in δ^18^O_planktonic_ and δ^13^C_*C.wueller*._; the deglacial temperate tree pollen record shows only subdued changes, a reflection of the small size of refugial tree populations. Of the seven intra-interglacial events, five show directly coupled pollen and δ^18^O_planktonic_ changes, while the local pollen minimum and δ^18^O_planktonic_ maximum are offset by one sample at the ~124.9 ka event. Only the ~117.2 ka event has negative δ^18^O_planktonic_ rather than positive values (although δ^13^C_*C. wueller*._ decreases).

### Comparisons with northern North Atlantic records

Farther north and in contrast to the NEEM LIG reconstructed surface temperature profile^19^, the most detailed North Atlantic records have revealed considerable variability. A series of cold water-mass expansions (cold events C28 to C25)^9^ has been reported at Ocean Drilling Project site 984 (ODP984) south of Iceland (Fig. 3e), coeval with changes at ODP980 on the Feni abyssal drift^8^. Reductions in North Atlantic Deep-Water production have been inferred from a series of δ^13^C_*C. wueller*._ and SST decreases in core MD03-2664 south of Greenland^10,38,39^ (Fig. 3f, g). The largest of these excursions is associated with event C27 and is characterized by the presence of a distinct red detrital layer, also detected in the Labrador Sea^40^ and attributed to an outburst flood through the Hudson Strait, analogous to the 8.2 ka event in the Holocene. A major meltwater discharge from the East Greenland margin^41^ may also be coeval with event C27.

Comparison of the Portuguese Margin and northern North Atlantic records reveals coherent changes in ocean surface conditions (Fig. 3). Large shifts in SST and IRD provide an unambiguous stratigraphic correlation of events HS11.1–HS11.3, C28, C25 and C24 between MD01-2444 and ODP984 and between ODP984 and MD03-2664; the temporal relations of intra-interglacial events C27, C27a, C27b and C26 (and one additional event C27′ observed in MD01-2444) are constrained by alignment of δ^18^O_planktonic_ and SST (see Methods and Supplementary Figs. 4, 5). These correlations, in turn, establish a link between North Atlantic cold events and southern European aridity events.

### Origin of LIG variability

The multi-centennial mid-to-high latitude North Atlantic surface coolings point to a significant reorganization of North Atlantic circulation. The cold water-mass expansions have been linked to southward displacements of the Arctic front^9,38^, but the trigger of these events remains unconstrained. Moderate freshwater input that increases stratification in the North Atlantic and leads to a weakening of the AMOC and attendant cooling in the North Atlantic represents one possible scenario. In addition to melting of MIS 6 ice sheets, LIG sea-level reconstructions require a contribution from the GrIS^3^. This is supported by marine sediment records off Greenland^42,43^, indicating GrIS retreat and runoff, especially in its southern sector. A series of large-amplitude decreases in δ^13^C_*C. wueller*._ in core MD03-2664 south of Greenland have been attributed to reductions in deep-water production^10^, driven by freshwater fluxes^40,42,43^. The δ^13^C_*C. wueller*._ records from shallower sites (ODP984 (ref. ^9^), ODP980 (ref. ^8^)) show smaller amplitude decreases and in some cases even small increases (in MD01-2444, Fig. 4d).

To investigate the impact of freshwater forcing in the North Atlantic during the LIG, we examine the spatial and temporal fingerprint of climate changes simulated by a series of climate-model experiments, using the Earth System model LOVECLIM^44^ under LIG boundary conditions (see Methods), which we compare to our palaeorecords. The model comprises an ocean general circulation model and a thermodynamic–dynamic sea-ice model, with a horizontal resolution of 3° longitude by 3° latitude and 20 vertical levels coupled to a spectral T21 quasi-geostrophic atmospheric model.

In our first experiment, 0.05 Sv of freshwater is added to the northern North Atlantic between 126.8 and 126.4 ka to represent event C27 (FWF005, see Methods), associated with freshwater discharges from residual ice sheets, including an outburst flood from the Laurentide ice sheet^10,40,41^. Events C27′ through C26 could have resulted from periods of enhanced GrIS melt, and are here represented by a more moderate freshwater input of 0.03 Sv between 124 and 123.6 ka in the northern North Atlantic (FWF003, see Methods). The total amount of freshwater added in these experiments corresponds to a rise of 1.78 m (4.4 mm yr^−1^) and 1.07 m (2.6 mm yr^−1^) sea-level equivalent (s.l.e.), for FW005 and FW003, respectively. To place these rates in the context of present changes, sea level has been rising by 3 mm yr^−1^ since 1993 as a result of accelerated melting of ice sheets and glaciers, and ocean thermal expansion^45^. It is important to note that while events C28 and C27 are still part of the penultimate deglaciation, the freshwater fluxes for the later events are within the upper estimates (3.5 m s.l.e.) of the GrIS contribution to global sea level^3^. More recently, combined ice-core surface temperature reconstructions and model simulations suggest a GrIS contribution of 5.1 m (4.1–6.2 m) and at least 3.9 m s.l.e. by 121 ka^46^. The smaller freshwater fluxes for C27′, C27a and C27b, with a combined total of ~3.2 m s.l.e., could represent periods of episodic Greenland meltwater discharges, but may have also included contributions from floating ice tongues, sea-ice or hydrological changes. Greenland ice regrowth after ~121 ka (refs. ^47,48^) would suggest that there was additional ice to melt for events C26 and C25.

**Fig. 5:**
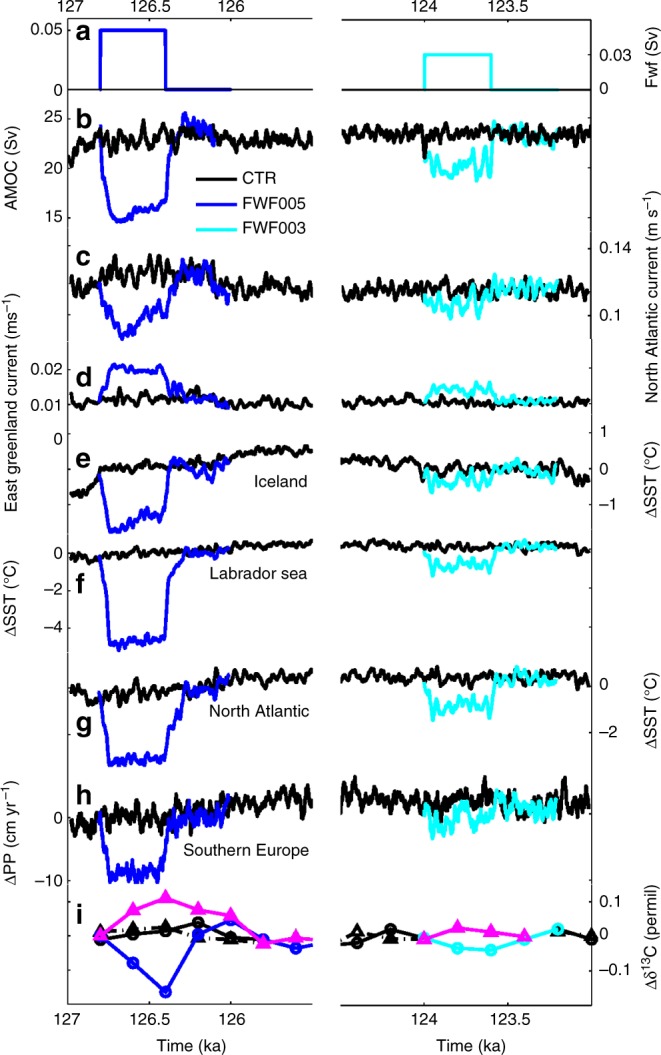
Results of a suite of LOVECLIM experiments performed under LIG conditions. Control experiment is shown in black and experiments where 0.05 Sv (FWF005) and 0.03 Sv (FWF003) of freshwater was added to the area south of Greenland for 400 years are shown in blue and cyan, respectively (see Methods). Time series of: **a** meltwater input in the North Atlantic; **b** maximum overturning circulation in the North Atlantic; **c** North Atlantic current strength (44°W–36°W, 45°N–47°N, 0–54 m); **d** East Greenland current (45°W–32°W, 56°N–68°N, 0–694 m); **e** SST anomalies averaged around Iceland (26°W–18°W, 60°N–64°N); **f** SST anomalies averaged around the Labrador Sea (60°W–40°W, 56°N– 60°N); **g** SST anomalies averaged around the North Atlantic (40°W–30°W, 50°N–58°N); **h** annual mean precipitation anomaly averaged over Southern Europe (10°W–20°E, 36°N–46°N); **i** δ^13^C anomalies averaged over 47°W–40°W, 47°N–51°N, 3300–4020 m (blue and cyan circles) and over 24°W–18°W, 56°N–62°N, 1443–1992 m (magenta triangles). To highlight the centennial-scale variability, time series **b** through **h** have been smoothed by a 21-year running mean

**Fig. 6:**
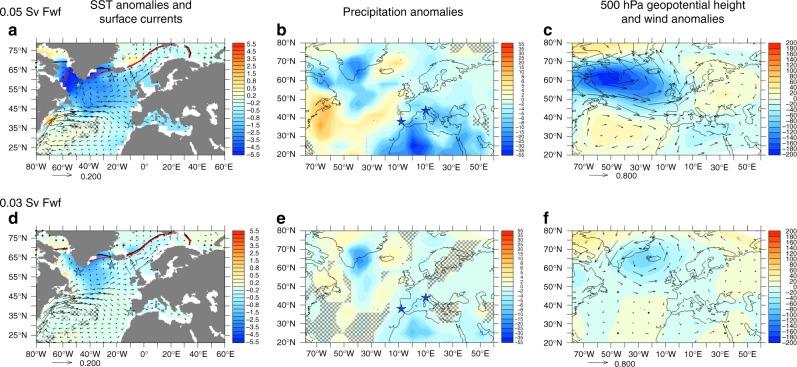
Climatic response to meltwater input in the North Atlantic. Results of a suite of experiments performed with LOVECLIM under LIG conditions and forced with (**a**–**c**) a 0.05 Sv and (**d**–**f**) 0.03 Sv meltwater input in the North Atlantic. Anomalies refer to annual mean changes averaged over (**a**–**c**) years 126.6–126.4 ka, and (**d**–**f**) years 123.8–123.6 ka compared to the control LIG state climate state. **a**, **d** SST anomalies (°C, shaded), 0.1 m sea-ice contour for the control LIG state (black) and perturbed state (red). Surface currents (m s^−1^, vectors) associated with the perturbed state are also shown. Stars show location of sites MD03-2664, ODP984 and MD01-2444. **b**, **e** precipitation anomalies (cm yr^−1^). Stars show location of MD01-2444 and Corchia Cave. The overlain grey grid indicates anomalies that are not statistically significant at the 95% level based on a Student *t* test. **c**, **f** Geopotential height (500 hPa) (m^2^ s^−2^, shaded) and winds (m s^−1^, vectors) anomalies

Within 100 years, the AMOC weakens by 35% and 20% in experiments FWF005 and FWF003, respectively (Fig. 5), reducing the advection of warm and salty waters to the Northeast Atlantic through the North Atlantic current, and inducing a significant cooling in the North Atlantic (Figs. 5e–g, 6a, d), in agreement with the SST^19^ and faunal records^9^. This surface cooling deepens the Icelandic Low, thus intensifying the subpolar gyre (SPG) and enhancing the advection of cold and freshwater through the East Greenland current. The overall surface temperature decrease in the North Atlantic and the associated reduced evaporation and changes in atmospheric circulation lead to a precipitation decrease over Southern Europe (Figs. 5h, 6b, e), in agreement with our pollen and speleothem records.

The link between North Atlantic cooling events and precipitation over southern Europe is further explored by forcing the atmospheric component of the NCAR CESM (see Methods) with estimates of North Atlantic surface cooling and extended sea-ice anomalies as simulated in experiments FWF005 and FWF003 (see Methods). The strong cooling over the northern North Atlantic leads to a deepening of the low-pressure system at mid-to-high latitudes, while the subtropical high-pressure system strengthens, extending towards Europe (Supplementary Fig. 6c, f). The anomalously strong anticyclonic circulation over Europe favours a more stable atmosphere and reduces convection, leading to drier conditions over the continent, particularly in its southern parts (Supplementary Fig. 6b, c). In addition, reduced ventilation of the deep North Atlantic, resulting from the AMOC weakening, leads to a significant δ^13^C decrease at depths below 3000 m in experiment FWF005 and a smaller decrease for the FWF003 experiment (Fig. 5i), consistent with the changes in palaeorecords^10^.

An alternative scenario for the origin of LIG variability is that changes in North Atlantic ocean surface circulation were driven by atmospheric processes. Observations over the past decades have highlighted the impact of North Atlantic westerly winds on the oceanic circulation and European climate associated with the North Atlantic Oscillation^49^. Modelling studies have further shown that this mode of variability leads to decadal changes in the SPG and in North Atlantic SST^50^. Through their modulation of the North Atlantic subtropical and SPGs, atmospheric processes may have thus been responsible for changes in SST in the past, and have been invoked to account for decadal to centennial changes in the SPG and oceanic reorganization in the North Atlantic during the past millennium^51^. Experiments with state-of-the-art coupled models using ~1000-year-long pre-industrial simulations have shown the occurrence of stochastic atmospheric changes in sea-level pressure pattern and winds, in one case enhancing sea-ice growth^52^, and in the other weakening the SPG^53^. These changes lead to a shutdown of deep-ocean convection in the Labrador Sea and a weakening of the AMOC for a period of 100–200 years. In these simulations, the persistent anomalies over high northern latitudes were possible through atmosphere, ocean and sea–ice interactions, with the weakening of the AMOC being a crucial factor in sustaining centennial-scale changes.

Thus, while different triggers may be hypothesized, that is, freshwater input or atmospheric forcing or a combination of the two, numerical simulations displaying centennial-scale cooling at the surface of the North Atlantic converge towards moderate AMOC weakening (of up to ~4 Sv).

### LIG vs. Holocene climate variability

**Fig. 7:**
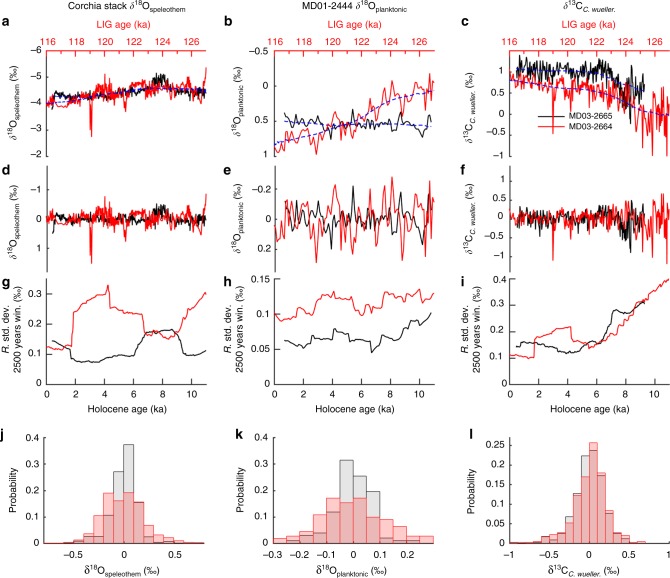
Comparison of Holocene and LIG variability (see Methods). **a**, **d**, **g**, **j** Corchia Cave δ^18^O_speleothem_ (Holocene record from stalagmite CC26 (ref. ^54^); LIG stack this study). **b**, **e**, **h**, **k** MD01-2444 δ^18^O_planktonic_ (Holocene record (ref. ^55^); LIG record, this study). **c**, **f**, **i**, **l** MD03-2664/5 δ^13^C_*C. wueller*._ (Holocene record from MD03-2665 (57°26.56′N, 48°36.60′W, 3400 m water depth (ref. ^56^); LIG record, this study). **a**–**c** Records resampled to an even time step with long-term trend represented by a 6000-year window Gaussian interpolation (dashed blue line). **d**–**f** Detrended records obtained by subtracting the long-term trend from resampled data. **g**–**i** Running standard deviations of the detrended records. **j**–**l** Relative frequency distributions of detrended records from Holocene (grey) and LIG (light red), where bar height equals number of observations in bin normalized to total number of observations (darker red indicates overlap). The Holocene and LIG intervals are overlain on the basis of aligning their insolation curves (Supplementary Fig. 7)

Finally, we compare the amplitude of LIG intra-interglacial variability with that of the Holocene, using records from the same sites with broadly similar sampling resolutions over the two intervals^54–56^. Figure 7a, b suggests that the amplitude of LIG excursions in the Corchia δ^18^O_speleothem_ and MD01-2444 δ^18^O_planktonic_ records is greater than in the Holocene. This is supported by examination of the running standard deviations and the relative frequency distributions of the detrended records, which isolate the centennial-scale intra-interglacial variability (see Methods). The running standard deviations of the detrended δ^18^O_planktonic_ and δ^18^O_speleothem_ records are consistently higher in the LIG compared to the equivalent Holocene section, except for the interval centred around 8 ka in the δ^18^O_speleothem_ record (Fig. 7g, h). Both δ^18^O_planktonic_ and δ^18^O_speleothem_ exhibit more extreme relative frequency distributions during the LIG as compared to the Holocene (Fig. 7j, k). Comparison of the δ^13^C_*C. wueller*._ records from adjacent sites MD03-2664 (ref. ^10^) and MD03-2665 (ref. ^56^), on the Eirik Drift, reveals overall more negative LIG values (Fig. 7c) and a long negative tail in the frequency distributions (Fig. 7l), consistent with reduced ventilation of the deep North Atlantic relative to the Holocene.

Could the warmer background state of the LIG have accentuated climate instability? State-of-the-art coupled model experiments under LIG boundary conditions are needed to test whether stochastic atmospheric forcing could trigger a series of events that are greater in amplitude than those of the Holocene. This is a possible scenario, but its explanatory power must be evaluated against the evidence for enhanced GrIS melting during the LIG compared to the Holocene^3,46^. Sediment data from south of Greenland indicate that, unlike the Holocene when GrIS runoff decreased at ~8 ka, the LIG GrIS retreat and runoff persisted throughout the interglacial until ~116 ka^43^. A series of anomalously low total air content values in air bubbles of the NEEM ice core from ~127 to 119 ka indicate multiple events of extensive surface melting^19^. Although the exact mechanism for repeated freshwater release from the GrIS is not clear, distinct peaks in titanium concentration from 127 to 116 ka in the sediment records off Greenland^57^ imply a series of pulses of increased runoff, albeit with uncertain magnitude.

Thus, while atmospheric and freshwater forcing are both consistent with moderate AMOC weakening, the palaeo evidence supports a role for episodic GrIS melting and runoff under intense boreal summer warming, contributing to the observed LIG variability. Whether intra-LIG AMOC disruptions were associated with changes in interhemispheric heat transport, for example, via a bipolar seesaw mechanism^58^, remains an open question. A LIG ultra-high-resolution δD record from the EPICA Dome C (EDC) ice core shows a series of low-amplitude intra-interglacial Antarctic warmings^59^. Timescale uncertainties, however, preclude a robust assessment of whether these are related to North Atlantic cold events or represent Antarctic ice sheet—Southern Ocean variability. Irrespective of these potential linkages, analysis of the variance of the EDC δD record indicates that climate conditions on the East Antarctic Plateau were systematically more variable during the LIG than the Holocene^59^, in agreement with our observations.

Accelerated freshwater input into the North Atlantic as a result of current global warming is expected to weaken the AMOC^60–62^. Though not a strict analogue for future anthropogenically driven changes, the profile of LIG that emerges is one of enhanced climate instability under conditions of ‘excess warmth’ relative to the pre-industrial Holocene, with implications for ice-sheet and ocean dynamics.

## Methods

### MD01-2444 core location and study interval

Core MD01-2444 (37°33.68′N; 10°08.53′W; 2637 m water depth; 27.45 m in length) was recovered near the location of core MD95-2042 (ref. ^20^) (37°48′N, 10°10′W; 3146 m water depth), in 2001, using the CALYPSO Giant Piston corer aboard the French research vessel *Marion Dufresne II*. This study focuses on the 22.50–19.50 m section in MD01-2444, (~140–108 ka), with a mean sedimentation rate of ~10 cm kyr^−1^. Samples for pollen and palaeoceanographic analyses were obtained from the same levels, typically at 1-cm intervals.

### MD01-2444 stable isotopes

Measurements were carried out in the Godwin Laboratory for Palaeoclimate Research (University of Cambridge) using a VG PRISM, VG SIRA, or Thermo MAT253 mass spectrometer. For PRISM/SIRA measurements, samples were dried in an oven at 50 °C overnight prior to sealing the vials with a septum and screw cap. The samples were analysed using a Micromass Multicarb Sample Preparation System attached to a VG SIRA or PRISM Mass Spectrometer. Each run of 30 samples is accompanied by 10 reference carbonates and 2 control samples. For the Thermo MAT253 measurements, foraminifera are transferred into sample vials, crushed and then dried in an oven at 50 °C. The vials were loaded into the carousel and analysed using a Thermo Kiel device attached to a Thermo MAT253 Mass Spectrometer in dual inlet mode. The preparation system operates automatically analysing samples in sequence. One hundred percent orthophosphoric acid is dropped onto the evacuated vial and reacts with the calcium carbonate sample. The evolved carbon dioxide is cryogenically dried and then admitted to the dual inlet mass spectrometer for isotopic analysis by comparison with a reference gas. Each run of 30 samples was accompanied by 10 reference carbonates and 2 control samples. The results are reported with reference to the international standard Vienna PeeDee Belemnite (VPDB) and the precision is better than ±0.06‰ for ^12^C/^13^C and ±0.08‰ for ^16^O/^18^O.

For the planktonic-isotope record about 15–30 specimens of *Globigerina bulloides* were selected from the 250 to 300 μm size fraction. Replicates were carried out for a selection of intervals using ~20–30 specimens from the 300 to 350 μm size fraction, confirming no apparent offset or differences in down-core variability in the different size fractions. Where replicate analyses were performed, measurements have been averaged.

For the benthic δ^18^O record, several different species (*Cibicidoides* sp., *Cibicidoides wuellerstorfi*, *Uvigerina peregrina*, *Hoeglundina elegans and Globobulimina affinis*) from the >212 μm size fraction were analysed as no single species was present in sufficient numbers to generate a continuous 1-cm sampling resolution record. Where possible two or three separate analyses of different species were made in each sample; a correction factor was applied (*Cibicidoides* sp.: +0.57; *U. peregrina* and similar specimens: 0.0; *G. affinis*: −0.3; *C. wuellerstorfi*, +0.64; *H. elegans*: −0.67). These adjustments are optimized for this particular core^63^ in accordance with the long-standing convention by which *U. peregrina* is assumed to deposit oxygen in isotopic equilibrium. The average of all the corrected values at each level is shown in the figures. The epibenthic δ^13^C record was derived from measurements on monospecific samples (1–4 individuals) of *C. wuellerstorfi* selected from the >212 μm size fraction wherever they were present. These new measurements were combined with those previously reported by ref ^25^.

Results are reported in Supplementary Data 1.

### MD01-2444 Mg/Ca measurements

Trace element measurements were conducted at the University of Cambridge via inductively coupled atomic emission spectrometry, using a Varian Vista machine^64^. New measurements have been combined with those previously reported^25^, with replicate analyses being averaged to yield a continuous record. All Mg/Ca analyses have been screened for dissolution artefacts by reference to shell weights and for contamination by reference to measured iron, aluminium and manganese concentrations. Only analyses with Fe/Ca and Mn/Ca ratios <0.5 mmol mol^−1^ were retained. Mg/Ca ratios in *G. bulloides* have been calibrated^65,66^ to temperatures, which yield absolute temperatures that correspond to the modern habitat ranges of these species. It must be stressed that any proxy record derived from a biological ‘proxy-carrier’ necessarily reflects a record of ‘habitat change’, and that this will reflect local climate change to the extent that the organism’s habitat has been coupled with the ‘synoptic’ local/regional climate. The ‘shallow water’ (here referred to as ‘surface water’, as distinct from ‘deep water’) temperature records derived here from *G. bulloides* Mg/Ca ratios thus represent a record of the impact of local climate change on the habitats of this planktonic foraminifer. At present, *G. bulloides* thrives near the surface under eutrophic (‘bloom’ or upwelling) conditions during spring and summer, or at frontal upwelling zones^67^.

The absolute uncertainty in the calculated temperature estimates (due to statistical calibration uncertainty and analytical reproducibility) is estimated at ~±0.7 °C. However, the ‘noise’ in the temperature time series, which can be approximated by the average standard deviation of paired adjacent measurements (which essentially provides an estimate of the degree of autocorrelation in the record), is closer to ±0.8 °C or about ±0.2 mmol mol^−1^. This estimate is obtained by assuming that, as their depth offset tends to zero, adjacent measurements will tend to represent replicates of a single mean value.

Estimates of seawater δ^18^O (δ^18^O_sw_) have been derived by combining calcite δ^18^O measurements with Mg/Ca-derived calcification temperature estimates, both performed on the same planktonic species. We use the palaeotemperature equation^68,69^, whereby:

1 $${\mathrm{T}}_{{\mathrm{Mg}}/{\mathrm{Ca}}} = 16.9 - 4.38\left( {\delta _{\mathrm{c}} - \delta _{\mathrm{sw}}} \right) + 0.1\left( {\delta _{\mathrm{c}} - \delta _{\mathrm{sw}}} \right)^2.$$

In the above equation $\delta _{\mathrm{c}}$ and $\delta _{\mathrm{sw}}$ are the calcite and seawater δ^18^O, respectively, and are both referenced to the same primary standard (i.e. $\delta _{\mathrm{c}}$ corrected by +0.2‰ to convert from VPDB to Vienna Standard Mean Ocean Water (VSMOW)).

Results are reported in Supplementary Data 1.

### MD01-2444 pollen analysis

Subsamples of 3–7 g of sediment were prepared for pollen analysis using the standard hot acid digestion technique. Fine sieving, through a mesh of 10 μm or less, was not used as it has been found to result in a loss of pollen, particularly Gramineae. Residues were mounted in silicone oil for microscopic analysis at magnifications of 400, 630 and 1000 times on a Leica DM2000 light microscope. Nomenclature follows ref ^70^. Abundances are expressed as percentages of the main sum, which includes all pollen except *Pinus*, Pteridophyte spores and aquatics. *Pinus* is conventionally excluded from the main sum, as it is strongly over-represented in marine sediments because of its extensive dispersal ability and buoyancy^71^. Following the convention for marine pollen analyses, a minimum of 100 pollen grains, excluding *Pinus*, spores and aquatics, was counted in each sample. Pollen totals (including *Pinus*) ranged from 123 to 540 grains. Shown here are the summary pollen curves of Mediterranean sclerophylls (*Olea*, *Pistacia*, *Phillyrea* and evergreen *Quercus*) and temperate trees, which includes Mediterranean sclerophylls and Eurosiberian taxa (the latter includes deciduous trees/shrubs and *Abies*, and excludes *Juniperus*, *Hippophae*, *Salix*, *Betula*). Results are reported in Supplementary Data 1.

Pollen studies from continental shelf sequences suggest that palynomorph transport to these areas is controlled primarily by fluvial and secondarily by aeolian processes^72^. Studies on modern pollen deposition in fluvial systems, considering the transfer of pollen from vegetation to the channel, transport in the channel and deposition in coastal waters, indicate the rapid incorporation of pollen to marine sediments^72^. At the Portuguese Margin margin, aeolian pollen transport is limited by the direction of the prevailing offshore winds and pollen is mainly transported to the abyssal site by the sediments carried by the Tagus River^73,74^. Comparison of modern marine and terrestrial samples along western Iberia has shown that the marine pollen assemblages provide an integrated picture of the regional vegetation of the adjacent continent^74^. In MD01-2444, the coherence of the pollen and marine isotope signals in MIS 3 and MIS 6 (ref. ^63^) argue against reworking of non-contemporaneous material.

### MD01-2444 XRF analysis

Archive halves of sections from Core MD01-2444 were analysed using an Avaatech XRF core scanner at the University of Cambridge^55^. The core surface was carefully scraped cleaned and covered with a 4-mm thin SPEXCertiPrep Ultralene foil to avoid contamination and minimize desiccation. XRF data were collected every 2.5 mm. The length and width of the irradiated surface was 2.5 and 12 mm, respectively, with a count time of 40 s. Results are reported in Supplementary Data 1.

### Corchia Cave analytical methods

The speleothem time series used in this study is derived from four Corchia Cave stalagmites (CC1, CC5, CC7 and CC28) collected from a single chamber (44°02′N, 10°18′E) between 1999 and 2005. The interval reported spans 140 to 108 ka. The cave setting, the collection details and the general characteristics of each stalagmite have been described in detail elsewhere^21,32,33,75,76^. Except for two small sections of CC5, each segment under study was microsampled at 100-μm resolution using a Taig CNC micromilling lathe. A ~41-mm interval of rapid growth in CC5 during the early LIG was sampled at intervals of between 0.2 and 2.0 mm, while another CC5 section (~38 mm length) at ~121 ka was sampled at an average interval of 0.12 mm.

The δ^18^O and δ^13^C measurements were conducted on two identical continuous-flow mass spectrometers at the Scottish Universities Environment Research Centre (SUERC) at East Kilbride, UK (an Analytical Precision *AP2003*) and the School of Earth Sciences, University of Newcastle, Australia (a GV Instruments *GV2003*). In both cases, subsamples of 0.8 ± 0.1 mg were digested in 105% orthophosphoric acid at 70 °C and measurements made on the evolved CO_2_. Sample results were converted to the VPDB scale using the known values of Cararra Marble house standards (NEW1 at Newcastle and MAB2C at SUERC) previously calibrated to the VPDB scale using NBS19 and NBS18. Each batch of 132 vials included between 22 and 27 house standards ~evenly spaced through the sequence. At Newcastle, scale corrections (small, <0.10‰) for δ^18^O were applied where necessary based on values obtained for two measurements each of NBS19 and NBS18. At SUERC, scale corrections were unnecessary, based on results of regular measurement of samples against both NBS19 and NBS18 using dual-inlet mass spectrometry (VG Prism II). The repeatability precision (1*σ*) on the AP/GV2003 machines throughout the analyses was ≤0.10‰ for δ^18^O and ≤0.05 for δ^13^C. Results are reported in Supplementary Data 2.

U–Th age data for each speleothem are shown in Supplementary Data 3. The U–Th dating was conducted either on samples drilled using a 1-mm drill bit or on composites of leftover powders originally microsampled for stable isotope analysis. The latter samples represented between 0.1 and 0.5 mm of speleothem distance (i.e. between one and five 100-μm-thick samples), depending on the amount of powder remaining. Conservative estimates were made of the 100% sampling uncertainty (Supplementary Data 3). Chemical separation of U and Th, spike procedures and the method of isotopic measurements followed the protocol of refs. ^77,78^. All age calculations use the latest U and Th decay constants^79^.

A Bayesian Monte Carlo method ‘finite growth-rate age model’ was used to calculate depth-age models for each stalagmite and to generate 95% age-uncertainty estimates for each stable isotope sampling depth position^21,32,80^. The depth-age plots and the modelled 2*σ* uncertainties for each speleothem are shown in Supplementary Fig. 1. Two U–Th ages were immediately rejected from CC5 because of their outlying position in depth-age space (red symbols in Supplementary Fig. 1; red text in Supplementary Data 3). Based on the updated U–Th age calculations, the time periods covered by each speleothem considered in this paper are:

CC1: 141.0 ± 1.6 to 128.5 ± 1.1 ka.

CC5: 141.0 ± 1.5 to 126.0 ± 1.0 ka, and 124.4 ± 0.99 to 107.0 ± 0.7 ka.

CC7: 128.6 ± 1.4 to 126.4 ± 1.5 ka, and 125.6 ± 1.3 to 121.3 ± 1.6 ka.

CC28: 118.4 ± 1.3 to 107.0 ± 1.0 ka.

### Corchia Cave composite (‘stacked’) time series

All but ~4 kyr of the 32-kyr interval is replicated and the isotopic patterns between coeval stalagmite sections display good consistency (not shown here). Accordingly, we constructed a single composite stable isotope record that attempts to maximize the level of detail by utilizing sections with the highest sampling resolution where possible, bearing in mind the challenges of splicing individual records into a single time series.

We used the stalagmite possessing the most complete record, CC5, as the tuning target. CC5 spans all but ~1.6 kyr of the 140–108 ka period. To produce the master time series, each individual speleothem depth series was first smoothed using a 5-point window. The stable isotopic data for CC1, CC7 and CC28 were then tuned to the CC5 depth scale using tie points common to overlapping growth segments. Tuning points were identified by eye using both δ^18^O and δ^13^C profiles. In difficult-to-match sections, the δ^18^O was used as the main tool for pattern matching, as this ratio is more robust than δ^13^C, which is more susceptible to local (including ‘in-cave’) influences.

For the long period of overlap between CC5 and CC28, we used the stable isotope data of the latter due to its superior time resolution. Data from this speleothem commence from a point as close as possible to its base where a clear isotopic matching with CC5 is evident. For the periods of overlap between CC7 and CC5, CC7 stable isotope data is only used across the hiatus in CC5 and where the time resolution is superior *and* there is confidence in the splice with CC5.

To develop the stack depth scale, the depth scale of CC5 was reset to an arbitrary zero corresponding to the termination of the longest period of continuous growth in this stalagmite (124.4–87.8 ka^32^), which occurs at an absolute depth position of 14.4 mm from the tip of CC5. To accommodate the hiatus in CC5 growth, a depth increment equivalent to the segment of the CC7 section was inserted into the stack depth scale. The pre-hiatus depth of CC5 was adjusted accordingly.

The results of the cross-tuning are shown in Fig. 2a, b and reveal good replication between the overlapping portions of the speleothems for both δ^18^O and δ^13^C. For CC7, we added 0.3‰ to the δ^18^O data to bring its profile into a more convincing alignment with CC5, although it appears this adjustment is somewhat inconsistent with the older segment. The need to apply an offset, and the inconsistency of such an offset over several thousand years, is not entirely surprising: the cave chamber lies at great depth below the ground surface (at least 400 m), and this plus the recharge elevation range of the structurally complex carbonate geology that comprises the aquifer^31^ would conspire to permit recharge waters from markedly different altitudes (and thus with different isotopic composition^31^) to reach the chamber at different times, according to the evolution of the flow path for individual drip points. The final stable isotope series on the stack depth scale is shown in Fig. 2b and reported in Supplementary Data 2.

The advantage of a single-stacked-record approach is that it allows the U–Th age data from *all speleothems* to be combined into a single depth-age model, thereby reducing overall age-model uncertainties for the entire 140–108 ka interval^21^. Accordingly, we transposed the original U–Th age-depth positions from the individual speleothems onto the stack depth scale and derived a new stack depth-age model using the Monte Carlo technique described previously (Fig. 2c, d). In implementing this model, we increased the sample-depth error (by ±1 mm) of all non-CC5 ages to account for uncertainty in the tuning procedure. The composite depth-age model reveals a further age outlier not detected in the original single-speleothem depth-age model, giving a total of three rejected outliers from a total of 90 U–Th age determinations. These outliers are highlighted by red symbols in Fig. 2c and have been excluded from the depth-age model.

Finally, a useful test of the robustness of the chronology of a stacked record is how closely the model stack age for each isotope data point agrees with its original model age. Supplementary Fig. 2 shows the results of this comparison. Only five very brief intervals display stack model ages outside the 95% uncertainties of the original model age, equivalent to ~2% of the entire record. In each case, the model-age difference is small (within 99% model-age uncertainties) and in no instance do these intervals correspond to the inferred aridity events identified in Fig. 3.

### MD01-2444 age model

The age model of the LIG section of MD01-2444 is based on aligning its temperate tree pollen curve to the δ^18^O_speleothem_ record from Corchia Cave (Supplementary Fig. 3), on the premise that, under the influence of mid-latitude westerlies and cyclogenesis over the Mediterranean, rainfall amount in southern Europe exerts a dominant control over both the composition of vegetation and isotopic signature of speleothems^21,31,34^. Supplementary Data 4 provide the alignment tie points, ages and associated uncertainties. It is important to emphasize that the comparisons of proxy records from the same samples in the MD01-2444 sediment sequence are independent of the choice of age model.

### Detrending the MD01-2444 and Corchia Cave records

To remove the low-frequency component from the temperate tree pollen, planktonic foraminifera and speleothem-isotope records, a 6-kyr Gaussian interpolation was subtracted from the data sets on their original time step. The residuals were then smoothed using a 600-year window Gaussian interpolation, significantly reducing the amplitude of periodicities below (shorter than) 400 years (Fig. 4).

### Comparison of intra-interglacial aridity events and ocean changes

To identify interglacial samples with negative temperate tree pollen and positive δ^18^O_planktonic_ values, the detrended data on their original time step (common to both data sets) were examined (Fig. 4). To avoid selecting minor variations that may be analytical noise or artefacts of the detrending method, temperate tree pollen and δ^18^O_plantonic_values in the same sample had to reach or exceed thresholds of –1.13% and +0.04 ‰, respectively. These thresholds are equivalent to one-third the mean absolute deviation of the respective data sets.

### Alignment of ODP984 to MD01-2444

The LIG record^9^ of ODP984 south of Iceland (61.25°N, 24.04°W; 1648 m water depth) is here aligned to that of MD01-2444 (Supplementary Fig. 4) on the basis that iceberg discharges, expansion of cold surface-water and attendant SST changes are quasi-synchronous (within the sampling resolution) between the two sites. Large changes in XRF Zr/Sr and IRD and also SST and percentages of *Neogloboquadrina pachyderma*, sinistral coiling (s.), considered a cold indicator species^9^, provide an unambiguous stratigraphic correlation of HS11.1–HS11.3 and C24. Smaller decreases in SSTs also constrain the correlation of C28 and C25. In between these events, the alignment of the two records is based on correlations of their respective δ^18^O_planktonic_ records. Supplementary Data 4 provide the alignment tie points, ages and propagated uncertainties.

### Alignment of MD03-2664 to ODP984

The LIG record^10,38,39^ of MD03-2664 from the Eirik Drift, south of Greenland (57°26.34′N, 48°36.35′W; 3442 m water depth) is here aligned to that of ODP984 (ref. ^9^) (Supplementary Fig. 5), on the basis that iceberg discharges, expansion of cold surface water and attendant SST changes are quasi-synchronous (within the sampling resolution) between the two sites. Large changes in IRD, SSTs and percentages of *N. pachyderma* (s.), provide an unambiguous stratigraphic correlation of HS11.1, HS11.2 and C25 and C24. Although the deglacial section of MD03-2664 is condensed, the end of HS11 and start of the LIG is clearly recorded in the IRD and SST records, which then allows the correlation of C28. The alignment of the intra-interglacial sections is based on correlations of changes in Mg/Ca SSTs in MD03-2664 and in the percentages of subpolar *N. incompta* percentages, representing a warm indicator species in ODP984 (ref. ^9^). Supplementary Data 4 provide the alignment tie points, ages and propagated uncertainties.

### Numerical experiments performed with LOVECLIM

North Atlantic meltwater experiments were performed with the Earth System model LOVECLIM^44^, which comprises a spectral T21 quasi-geostrophic atmospheric model and an ocean general circulation model coupled to a thermodynamic-dynamic sea-ice model, with a horizontal resolution of 3° longitude by 3° latitude and 20 vertical levels. The experiments were performed under varying LIG boundary conditions, that is, appropriate orbital parameters^81^, Northern Hemisphere ice-sheet extent and albedo^82^, atmospheric CO_2_ content^83^ and constant CH_4_ (700 ppb) and N_2_O (310 ppb) values. Two experiments were performed with meltwater added in the northern North Atlantic, to the area south of Greenland (55.5°N–66.3°N, 58.5°W–31.5°W) for 400 years. For the first experiment, 0.05 Sv (10^3^ m^3^ s^−1^) was added between 126.8 and 126.4 ka (FWF005), and for the second one, 0.03 Sv was added between 124 and 123.6 ka (FWF003). Both experiments run for another 400 years without any forcing and were compared to an LIG control experiment. Following the freshwater addition, the AMOC weakens from ~23 to 15 Sv (~35%) at 126.7 ka and from 24 to 19 Sv (~20%) at 123.9 ka. We note that LOVECLIM’s North Atlantic and European climate response to an AMOC weakening is within the range of coupled climate models^84^.

### Numerical experiments performed with CESM

To further assess the link between changes in North Atlantic SST and precipitation over Europe, we perform additional experiments with an independent model, the NCAR CESM. The atmospheric component of the CESM, namely the Community Atmospheric Model version 4 (ref. ^85^) is forced with monthly SST and sea-ice conditions from LOVECLIM. The CAM4 configuration consists of a spatial resolution of 1.9° latitude by 2.5° longitude and 26 vertical levels in a hybrid sigma-pressure coordinate. Four experiments are performed with an atmospheric CO_2_ concentration fixed at 284.7 ppmv and integrated as follows: (i and ii) two control run experiments forced with a repeating 12-month climatology of SST and sea-ice concentration from the LOVECLIM LIG control run at 126.8 and 124 ka; (iii) an experiment forced with a climatology of both North Atlantic SST and sea-ice concentration derived from the LOVECLIM FWF005 simulation when the AMOC is reduced by 35% (Supplementary Fig. 6b, c); and (iv) an experiment forced with a climatology of both North Atlantic SST and sea-ice concentration derived from the LOVECLIM FWF003 simulation when the AMOC is reduced by 20% (Supplementary Fig. 6e, f).

Outside the forcing domain, a 10° latitude/longitude band was used to linearly damp the North Atlantic SST and sea-ice conditions to the control run climatology. Unless otherwise specified, SST and sea-ice concentration were the same as for the control run. The control runs were integrated for 270 and 100 years and the perturbation experiments were integrated for 90 and 80 years, respectively.

### Analysis of the variance of LIG and Holocene records

Holocene and LIG Corchia Cave δ^18^O_speleothem,_ MD01-2444 δ^18^O_planktonic_ and MD03-2664/5 δ^13^C_*C. wueller*._ were each linearly resampled to an even time step (δ^18^O_speleothem_ = 10 years, δ^18^O_planktonic_ = 100 years, δ^13^C_*C. wueller*._ = 25 years) (Fig. 7a–c) and detrended by removing a 6000-year Gaussian interpolation (Fig. 7d–f). No smoothing was applied to the detrended data. To compare the relative variability of Holocene and LIG records, two approaches were used. First, a 2500-year window running standard deviation was calculated for each record (Fig. 7g–i). The 2500-year window is centred, meaning that the first and last 1250 years of the running standard deviations have different statistical properties to the remaining data. Second, histograms were constructed to compare the relative frequency distributions of the Corchia δ^18^O_speleothem,_ δ^18^O_planktonic and_ δ^13^C_*C. wueller*._ detrended records in the Holocene and LIG. Bin width is consistent for Holocene and LIG records and bar height equals number of observations in bin normalized to total number of observations (Fig. 7j–l).

## Electronic supplementary material

Supplementary Information

Description of Additional Supplementary Files

Supplementary Data 1

Supplementary Data 2

Supplementary Data 3

Supplementary Data 4

## Data Availability

All data generated for this study are available in the article and its Supplementary materials.
