# Supplementary material for: Enhanced climate instability in the North Atlantic and southern Europe during the Last Interglacial
Source: Nat Commun. 2018 Oct 12;9:4235. doi: 10.1038/s41467-018-06683-3 (PMC6185935; doi:10.1038/s41467-018-06683-3)
Supplement: Supplementary file 1 — Supplementary Information [file 41467_2018_6683_MOESM1_ESM.pdf]

Supplementary information for

Enhanced climate instability in the North Atlantic and southern Europe during the Last Interglacial

P. C. Tzedakis et al.

Correspondence to: p.c.tzedakis@ucl.ac.uk

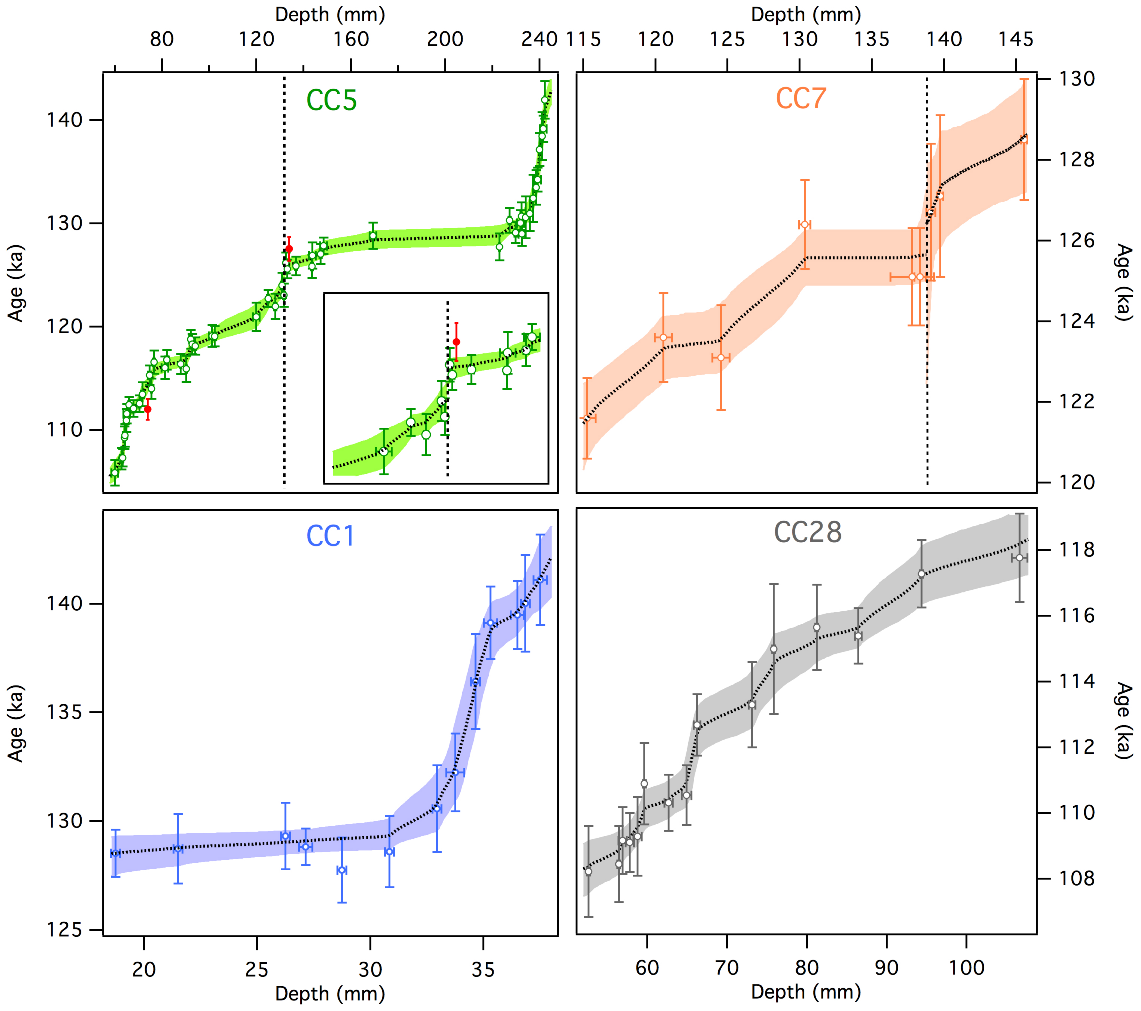

Supplementary Fig. 1. Depth-age models for stalagmites CC1, CC5, CC7 and CC28 from Corchia Cave for the period under investigation in this study. Vertical dashed lines in the CC5 and CC7 plots mark the position of hiatuses. Sampling (horizontal- 100%) and analytical (vertical- 95%) errors bars are shown. In the CC5 plot, the inset is an enlargement of the region around the hiatus. Red symbols in CC5 are outliers rejected for that stalagmite's depth-age model.

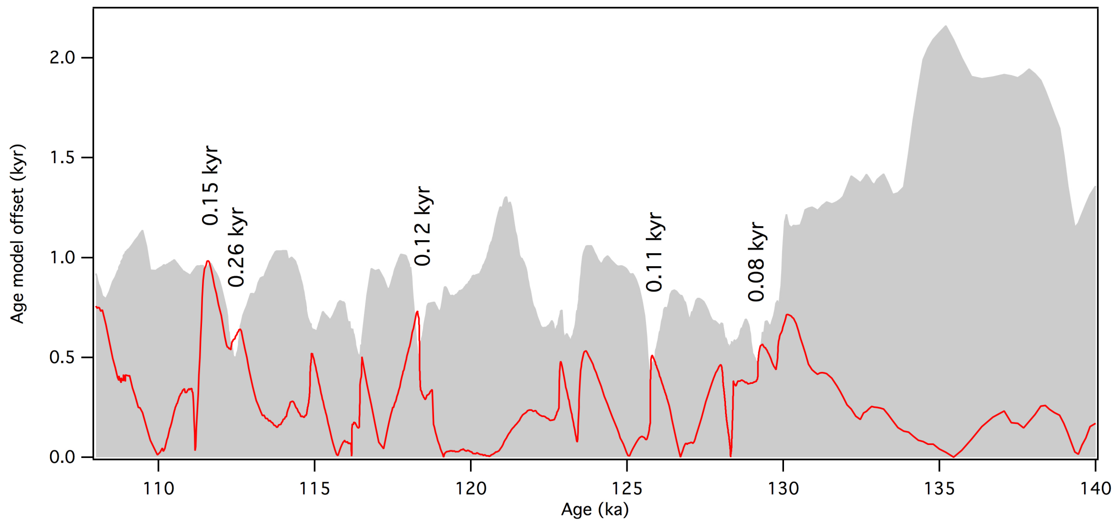

Supplementary Fig. 2. The difference (in kyr) between the original model age and the stack model age for each data point used in the Corchia Cave stable isotope stack (red line). The grey shaded area is the original model-age uncertainty of the same data points. Segments where the red line exceeds the grey shaded area correspond to intervals where the new age lies outside the 95% uncertainties of the original age. The numbers are the durations of these intervals, which are very brief (2.25% of the whole record) and do not clash with the inferred aridity events identified in Fig. 2.

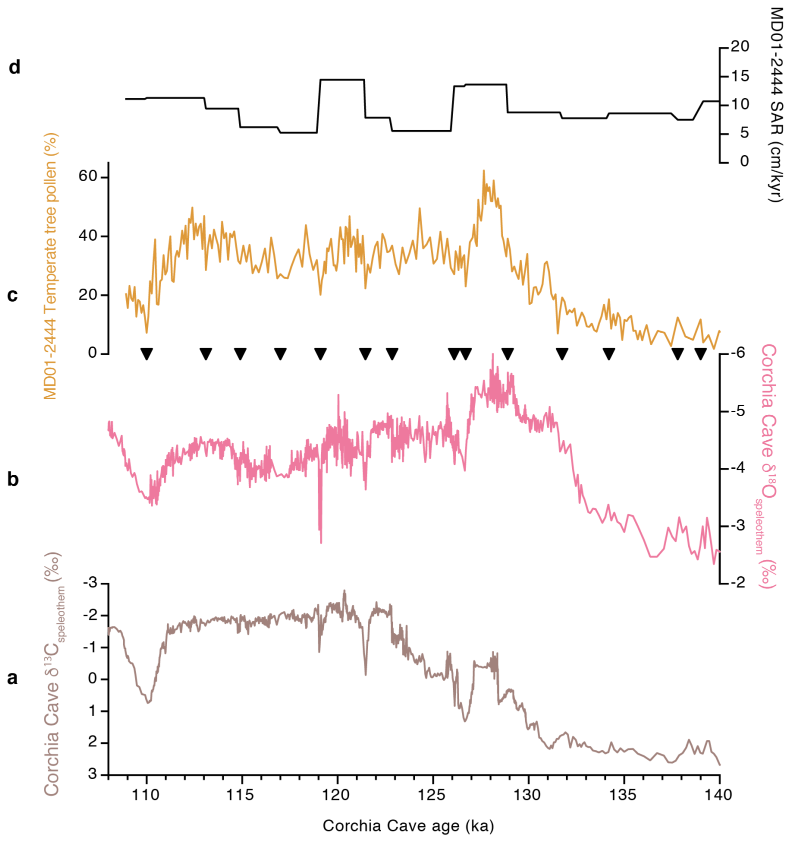

Supplementary Fig. 3. Alignment of MD01-2444 temperate tree pollen record to Corchia $\delta^{18}O_{speleothem}$ record. **a**, Corchia Cave stacked speleothem $\delta^{13}C$ series. **b**, Corchia Cave stacked speleothem $\delta^{18}O$ series. **c**, MD01-2444 temperate tree pollen percentages. **d**, MD01-2444 sediment accumulation rates. Tie points are indicated by triangles.

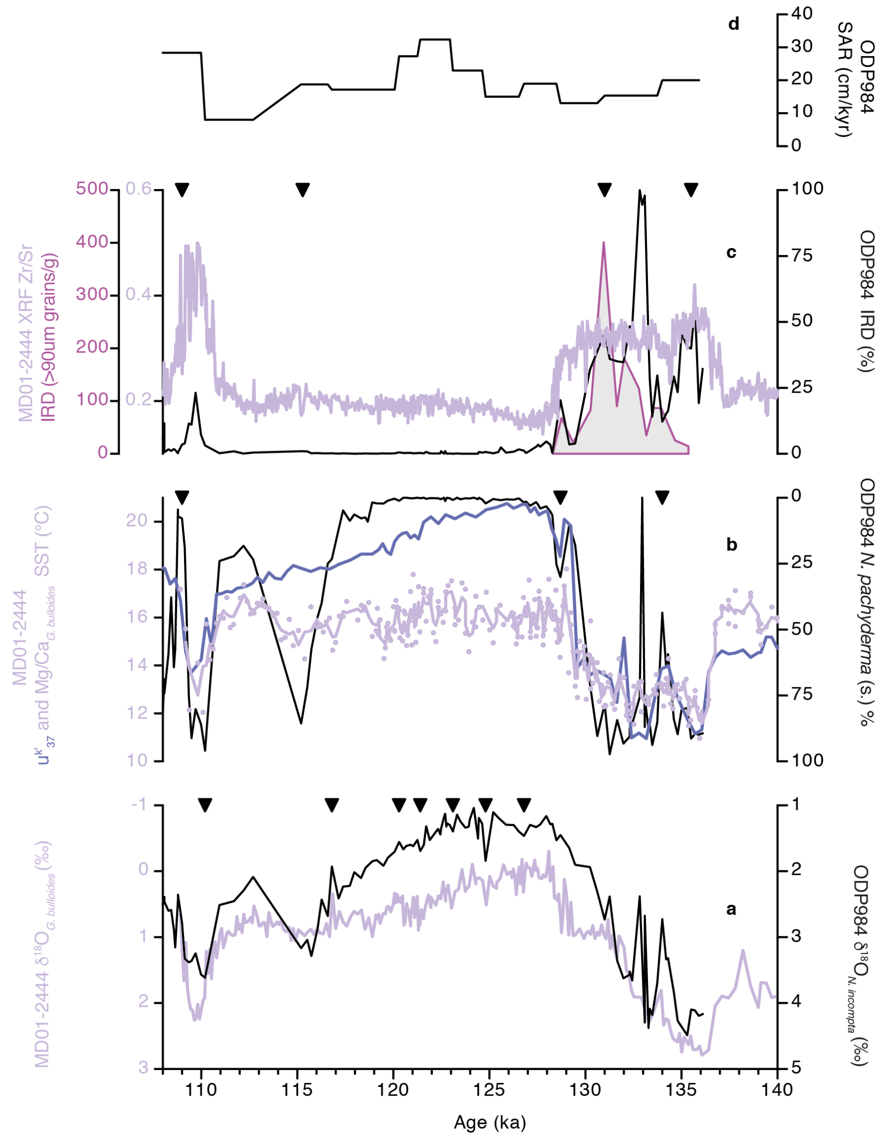

Supplementary Fig. 4. Alignment of ODP984 (ref. 1) to MD01-2444. a, MD01-2444 (mauve) and ODP984 (black) planktonic $\delta^{18}O$ records. **b,** MD01-2444 SST (mauve: Mg/Ca; blue: $U^{k'}_{37}$) *versus* ODP984 *N. pachyderma* (s) abundance (black, inverse scale). **c,** MD01-2444 XRF Zr/Sr (mauve), and IRD percentages in MD01-2444 (red violet outline; counts for interval 21.30-21.95 m only) and ODP984 (black). **d,** ODP984 sediment accumulation rates. Tie points are indicated by triangles.

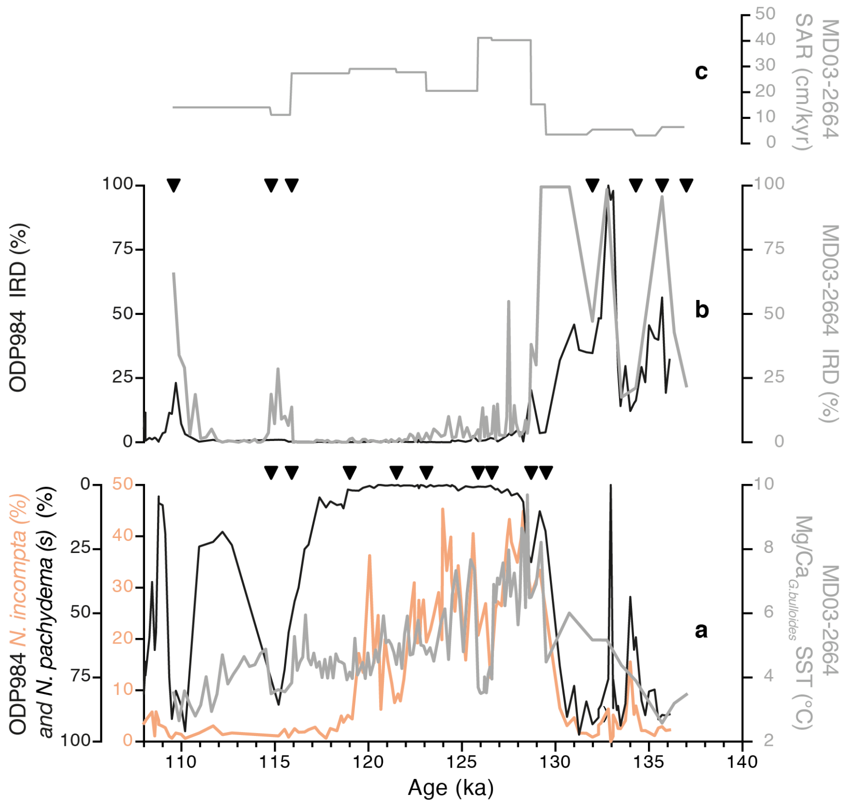

Supplementary Fig. 5. Alignment of MD03-2664 (refs 2-4) to ODP984. a, ODP984 *N. incompta* (orange) and *N. pachyderma* (s) (black; inverse scale) abundances *versus* MD03-2664 SST. **b,** IRD percentages in ODP984 (black) and MD03-2664 (grey). **c,** MD03-2664 sediment accumulation rates. Tie points are indicated by triangles.

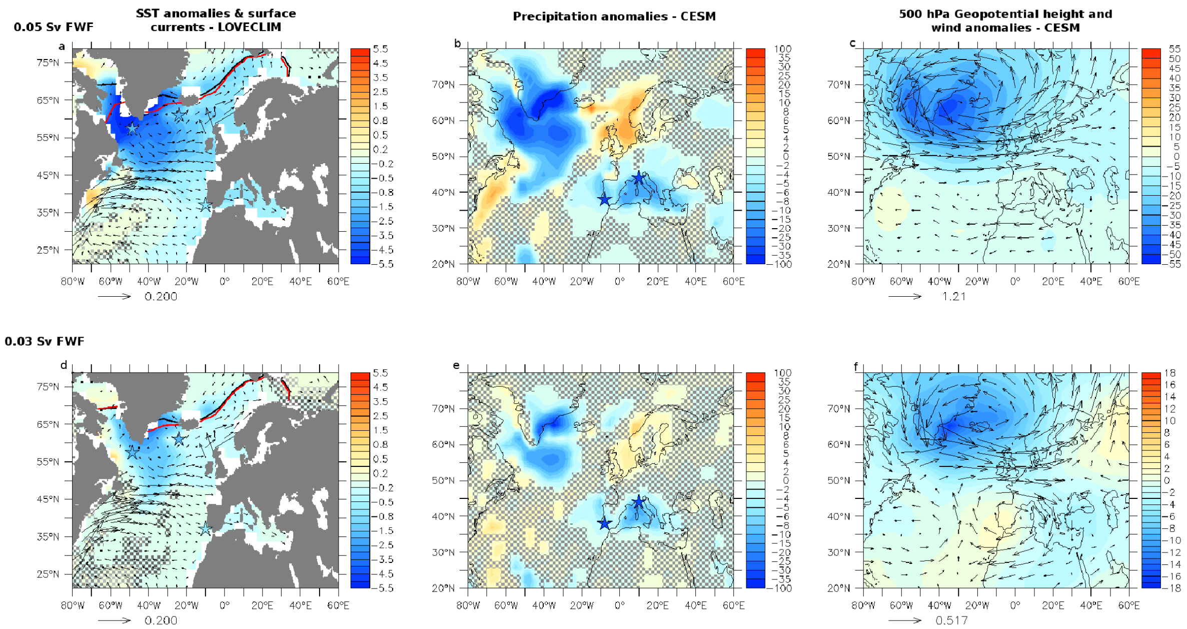

Supplementary Fig 6. **Results of experiments performed with the atmospheric component of CESM**. **a and d,** annual mean SST anomalies, surface currents and 0.1m sea-ice contour as simulated by LOVECLIM. Stars show location of sites MD03-2664, ODP984 and MD01-2444. **b and e,** annual mean precipitation anomalies (cm yr^{-1}, grid indicates non-significant anomalies). Stars show location of MD01-2444 and Corchia Cave. **c and f,** geopotential height and wind anomalies at 500 hPa as simulated by CESM for experiments forced with a 0.05 Sv (**a-c**) and a 0.03 Sv (**d-f**) North Atlantic freshwater flux.

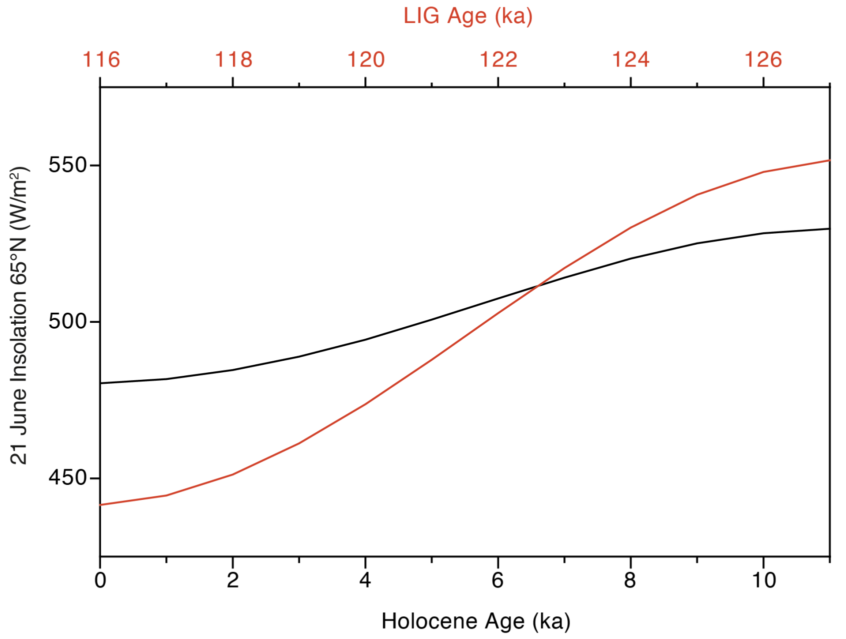

Supplementary Fig 7. Alignment of Holocene and LIG insolation records. Shown are mean daily insolation on 21 June at 65°N (ref. 5) records for the Holocene (black) and LIG (red). The two curves are aligned at their respective maxima (11 and 127 ka).

Supplementary References

1. Mokeddem, Z., McManus, J. F. & Oppo, D. W. Oceanographic dynamics and the end of the last interglacial in the subpolar North Atlantic. *Proc. Natl. Acad. Sci. U.S.A.* **111**, 11263-11268 (2014).
2. Galaasen, E. V. et al. Rapid reductions in North Atlantic Deep Water during the peak of the last interglacial period. *Science* **343**, 1129-1132 (2014).
3. Irvali, N. et al. Rapid switches in subpolar North Atlantic hydrography and climate during the Last Interglacial (MIS 5e). *Paleoceanography* **27**, PA2207 (2012).
4. Irvali, N. et al. Evidence for regional cooling, frontal advances, and East Greenland Ice-sheet changes during the demise of the last interglacial. *Quat. Sci. Rev.* **150**, 184-199 (2016).
5. Laskar, J. et al. A long-term numerical solution for the insolation quantities of the Earth. *Astron. Astrophys*. **428**, 261–285 (2004).
