# Supplementary material for: Enhanced climate instability in the North Atlantic and southern Europe during the Last Interglacial
Source: Nat Commun. 2018 Oct 12;9:4235. doi: 10.1038/s41467-018-06683-3 (PMC6185935; doi:10.1038/s41467-018-06683-3)
Supplement: Supplementary file 2 — Description of Additional Supplementary Files [file 41467_2018_6683_MOESM2_ESM.pdf]

Description of Additional Supplementary Files

File Name: Supplementary Data 1
Description: MD01-2444 data from palaeoceanographic, sediment and pollen analyses.

File Name: Supplementary Data 2
Description: Corchia Cave isotopic data

File Name: Supplementary Data 3
Description: Corchia Cave U-Th age data

File Name: Supplementary Data 4
Description: Age models for MD01-2444, ODP984 and MD03-2664
